# Hemifacial spasm caused by unruptured fusiform vertebral aneurysm treated with endovascular coil embolization: a case report

**DOI:** 10.3389/fneur.2023.1203751

**Published:** 2023-07-25

**Authors:** Pengchen He, Zongping Li, Han Jiang

**Affiliations:** ^1^Department of Neurosurgery, Mianyang Central Hospital, School of Medicine, University of Electronic Science and Technology of China, Mianyang, Sichuan, China; ^2^Department of Rehabilitation Therapy, Mianyang Central Hospital, School of Medicine, University of Electronic Science and Technology of China, Mianyang, Sichuan, China

**Keywords:** hemifacial spasm, occlusion, vertebral artery, fusiform aneurysm, lateral spread response

## Abstract

Hemifacial spasm due to fusiform aneurysm of the vertebral artery is extremely rare. The lateral spread response (LSR) is routinely used to monitor hemifacial spasms during microvascular decompression to predict the degree of postoperative remission of hemifacial spasm. We report a case of hemifacial spasm caused by an unruptured fusiform vertebral aneurysm treated with intravascular intervention and monitoring of LSR. A 59-year-old man was admitted to the hospital with a left facial spasm that gradually worsened for 1 year. Preoperative cerebrovascular angiography indicated fusiform aneurysms in the intracranial segment of the left vertebral artery close to the left facial nerve. The patient underwent parent artery occlusion and aneurysm embolization, and LSR was monitored intraoperatively. After intraoperative aneurysm embolization, LSR disappeared immediately. The postoperative review of cerebrovascular angiography indicated that the parent artery and aneurysm were embolized successfully, and the patient's left facial spasm was relieved after surgery. Hemifacial spasm caused by the vertebral artery fusiform aneurysm can be safely and effectively treated by parent artery occlusion and aneurysm embolization. Meanwhile, intraoperative LSR monitoring can be used to predict postoperative efficacy.

## 1. Introduction

Hemifacial spasm (HFS) is typically caused by the curvature and elongation of the facial nerve into the brain stem root exit zone (REZ) and branches of the vertebrobasal nervous system or the vertebral artery (VA). Microvascular decompression (MVD) is the most effective hemifacial spasm treatment (1, 2). The LSR is widely used to guide MVD and predict postoperative efficacy as an objective electrophysiological monitoring indicator of HFS (3). The occurrence of HFS caused by vertebral fusiform artery aneurysms is extremely rare. A case of HFS caused by an ipsilateral fusiform vertebral artery aneurysm is described. In the meantime, intraoperative electrophysiological monitoring was used to record changes in LSR before and after intervention to predict postoperative efficacy.

## 2. Case report

**Figure 1:**
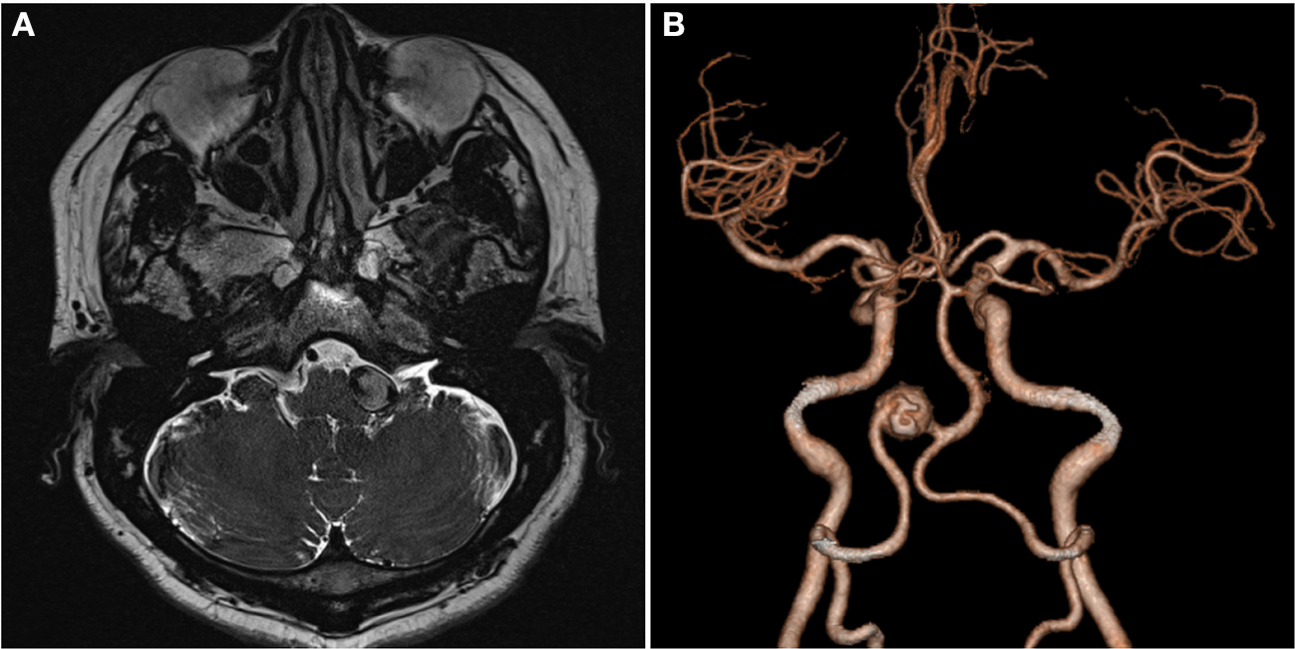
Images of the patient upon admission. **(A)** Axial T2-weighted MR image shows a fusiform aneurysm as a flow void signal left of the pons. **(B)** Three-dimensional CT angiography shows VA fusiform aneurysm. VA, vertebral artery.

**Figure 2:**
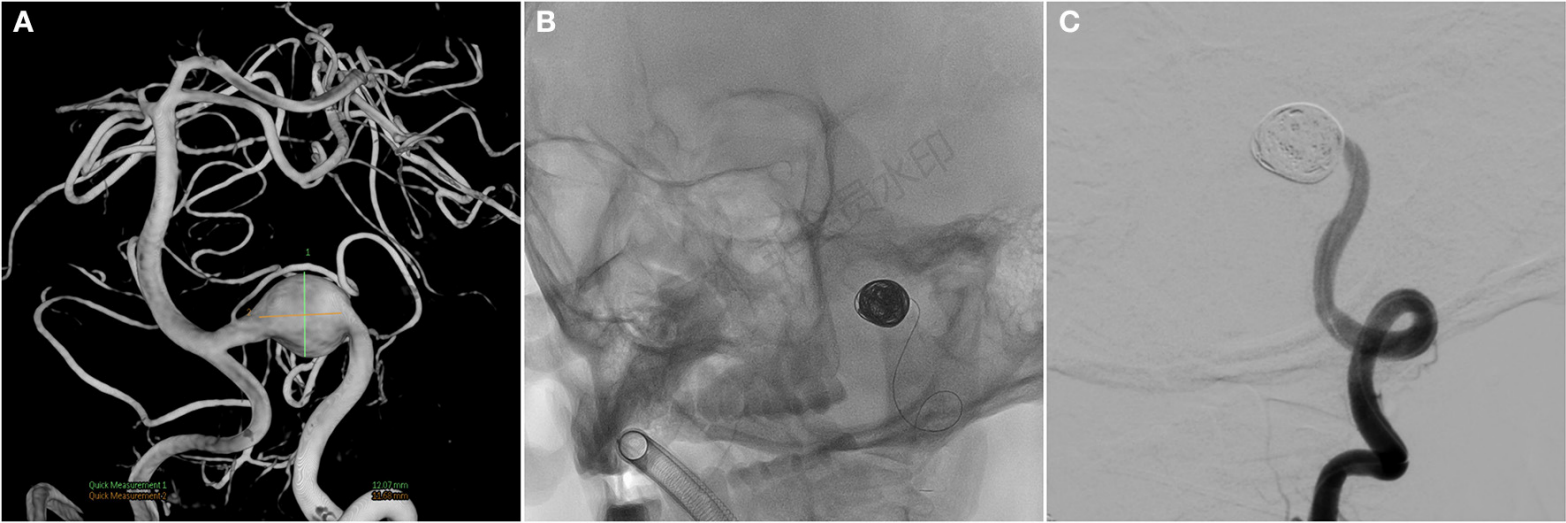
**(A)** Cerebral angiography before and after endovascular treatment and preoperative cerebral angiogram shows a fusiform aneurysm at the V3 segment. **(B, C)** Angiograms after endovascular treatment show the complete occlusion of left VA, including aneurysm. VA, vertebral artery.

A 59-year-old man presented with paroxysmal involuntary spasm in the left orbital area that gradually worsened 1 year ago before admission to our hospital. After admission, three-dimensional CT reconstruction examination of the intracranial artery showed that the intracranial segment of the left vertebral artery was locally enlarged and nodular, with a size of ~1.5 cm × 1.3 cm. Magnetic resonance imaging revealed an aneurysm of the intracranial segment of the left vertebral artery, which was appressed to the left facial nerve (Figure 1). Cerebral angiography revealed an aneurysmal enlargement in the V3 segment of the left vertebral artery, with no obvious aneurysm neck and a maximum transverse diameter of 1.2 cm (Figure 2A). No other possible causative lesions for the hemifacial spasm were identified.

**Figure 3:**
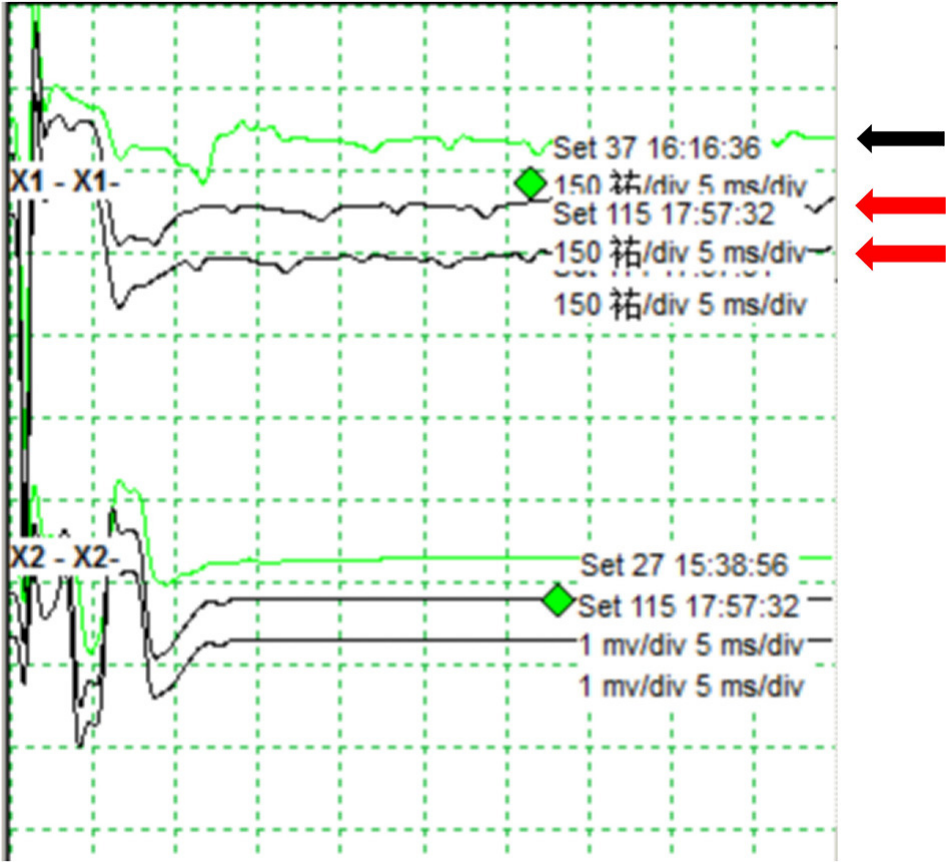
Monitoring of the lateral spread response during endovascular surgery. The LSR shows before embolization of the aneurysm (black arrow). The LSR disappears after intraoperative embolization of the aneurysm (red arrow).

Because the aneurysm is fusiform and the parent artery is tortuous, it is difficult to clip the aneurysm directly during craniotomy. Concurrently, there was a certain distance between PICA and aneurysm, so PAO was selected. We intended to use parent artery occlusion (PAO) and coil embolization during surgery after evaluating our patients. The patient was given the following medications as preoperative and perioperative measures: oral aspirin 100 mg/day for 3 days before surgery with clopidogrel 75 mg/day, intraoperative tirofiban acid injection for arterial thrombosis prevention and papaverine hydrochloride injection heparin 80IU/Kg were given intravenously before the operation to prevent cerebral vasospasm, and then, half heparin was added every hour until 1,000 IU was maintained. The patient was then operated under general anesthesia. A unilateral femoral artery was punctured by the Seldinger method, a 6F guide sheath was placed, and a 5F angiography catheter was placed. The internal carotid artery and vertebral artery were overselected for standard angiography, and then, 3D-DSA was performed on the left vertebral artery. The 5F guide catheter (Johnson & Johnson, USA) was superselected for the V2 segment for further angiography, and PAO was performed after the indications were clear. An Excelsior SL-10 microcatheter (Stryker, USA) was superselected through the ipsilateral vertebral artery under the guidance of a microguide wire into the aneurysm, and seven coils (MicroVention, USA) were used to fill the aneurysm completely (Figures 2B, C). 2 hours after the surgery, the sheath was removed, and the femoral artery puncture point was pressed for 15 min, the elastic bandage “8” word compression bandage could be used when there was no active bleeding, total bed rest, and the lower limb was immobilized for 12 h. The marginal mandibular branches of the facial nerve were stimulated with fixed frequency and intensity, the LSR was recorded in the mentalis and orbicularis oculi muscle, and the LSR was monitored throughout the operation. After intraoperative embolization of the aneurysm, electrophysiological monitoring showed that LSR disappeared (Figure 3). Postoperative DSA examination indicated complete embolization of the aneurysm and parent artery, and the patient's left facial spasm was significantly relieved. A month later, the patient's symptoms completely disappeared.

To prevent thrombosis and ischemic events after surgery, the patient was instructed to take aspirin 100 mg/d and clopidogrel 75 mg/d orally the following day. Clopidogrel was discontinued 6 weeks later, and asprin was prescribed for life for a year.

## 3. Discussion

HFS is a functional neurological disease characterized by involuntary spasms of the muscles innervated by the facial nerve and its branches, which is mainly caused by the direct compression of the REZ area by the branch of the vertebrobasilar system or the curvature and elongation of the vertebral artery (VA) itself (1). HFS can also be caused by rare aneurysms, arteriovenous malformations, and tumors in the cerebellopontine angle. HFS caused by fusiform vertebral aneurysm is extremely rare. Four cases have been reported to date (Table 1) (11, 12). Most vertebral saccular aneurysms with HFS can be clipped by craniotomy, and the REZ region can be explored at the same time to separate the aneurysm from the adhesion of the facial nerve root, which can cure both aneurysm and HFS (11, 13). However, because most vertebral aneurysms are fusiform and direct clipping is difficult, the majority of them were treated with isolated and interventional embolization (14). The patient in this case had a fusiform aneurysm at the V3 segment of the left vertebral artery, and imaging revealed that the aneurysm was fusiform with no clear aneurysmal neck and a large aneurysm volume. Endovascular treatment was chosen due to the simultaneous distortion of the vertebral artery and the proximity of the aneurysm to the brain stem and vulnerable cranial nerves.

**Table 1:** Summary of reports of symptomatic hemifacial spasm caused by aneurysm of the vertebral artery.^α^

**Author (year)**	**Age, sex**	**Side**	**Type**	**Period of HFS**	**Size of aneurysm (mm)**	**Treatment**	**LSR**	**Decomp**	**Outcome**
Moriuchi et al. (4)	62, F	Left	Saccular	6 years		Neck clipping	-	+	Disappeared immediately
Tsuchiya et al. (5)	71, F	Right	Fusiform	11 years	-	Neck clipping	-	+	Disappeared immediately
Sato et al. (6)	53, M	Left	Saccular	2 years	15 × 8 × 4	Coil embolization	-	-	Disappeared 6 months later
Murakami et al. (7)	49, F	Right	Fusiform	9 months	-	Coil embolization	+	-	Disappeared 6 months later
Uchino et al. (8)	59, M	Left	Fusiform	18 months	-	MVD only	-	+	Disappeared immediately
Nakagawa et al. (9)	55, F	Left	Fusiform	2 years	13	Coil embolization	-	-	Disappeared 3 months later
Lee et al. (4)	69, M	Left	Fusiform	5 years	-	Extracranial VA ligation	-	+	Disappeared immediately
Iida et al. (10)	59, M	Left	Saccular	2 months	5.5	Coil embolization	-	-	Disappeared immediately
Our case	59, M	Left	Fusiform	1 year	15 × 13	Coil embolization	+	-	Disappeared 1 month later

^α^F, female; M, male; Decomp, decompression; HFS, hemifacial spasm; LSR, lateral spread response.

The primary endovascular interventions for fusiform vertebral artery aneurysms are PAO and parent artery reconstruction (PAR). Before surgery, PAO may need to perform a balloon occlusion test (BOT) to confirm the compensation of the contralateral vertebral artery to the basilar artery or ipsilateral PICA, as well as good blood supply from anterior to posterior circulation. Most patients are candidates for PAO. PAO has the advantage of improving the degree of distal aneurysm embolization, reducing the number of coils, and alleviating the mass effect. It can be used to treat cranial nerve compression caused by long lesion segments, irregular shapes, or large aneurysms. However, proximal PICA occlusion is easily caused, resulting in a significant increase in the incidence of ischemic events, especially when the lesion involves PICA or the diseased vessel is the basilar artery's dominant supplying artery. PAR is appropriate for patients who are unable to tolerate BOT tests, have a small aneurysm, and are not expected to rupture prematurely (15, 16). According to the preoperative imaging examination, the aneurysm tumor was fusiform and larger than 1.5 cm in size, and the PICA measured during DSA was approximately 1 cm away from the distal end of the aneurysm. PAR may cause ischemic events in the PICA or other perforator vessels in the long run. In contrast, PAO is less likely to cause ischemic events. As a result, PAO is chosen to simultaneously embolize aneurysms. During the procedure, a single catheter was used to gradually release the coil. Open surgery was another option for this patient. Direct clipping is exceedingly challenging in this situation because there is not a definite neck of the aneurysm. Tsunoda et al. (17) reported a case of surgical removal using V3-radial artery graft-V4 bypass and occipital artery-posterior inferior cerebellar artery bypass for a giant thrombosed aneurysm. In this approach, the complete restoration of the distal vertebral artery blood flow is consistent with the physiological function of the human body. While the procedure is being performed, the connection between the cranial nerve and aneurysm can be investigated, as well as any potential involvement of further vessels. However, endovascular techniques are believed to be less intrusive than open surgical manipulation. The risk of surgery was particularly enhanced by the size of the aneurysm and its near proximity to peripheral nerves and blood vessels in this case. Meanwhile, another drawback of surgery is that it takes a long time and is extremely complicated and invasive.

When the zygomatic branch of the facial nerve is stimulated in patients with HSF, an abnormal electrical change in the other branches is detected, which is known as LSR. The mechanism by which it occurs is unknown (18). The compression of responsible blood vessels, demyelination of facial axons, and formation of reverse afferent stimuli via pseudosynapses all contribute to the production of LSR. When the responsible vessel is separated from the nerve, the LSR should vanish immediately, allowing the effectiveness of MVD to be monitored. However, because HFS is rarely caused by the aneurysm itself, the mechanism of HFS remission following endovascular treatment of aneurysms remains unknown. Nakagawa et al. (9) reported a patient with HFS caused by a contralateral fusiform aneurysm, whose symptoms improved gradually 3 months after aneurysm interventional embolization. The gradual improvement in the aberrant excitatory circuit at the REZ may parallel the gradual decrease in facial nerve compression following intravascular treatment of the aneurysm, alleviating the hemifacial spasm. The reduction of the aneurysm mass effect was attributed to the remission of HFS symptoms. Similarly, the relief of ophthalmic paralysis after endovascular therapy for cavernous sinus aneurysm and the relief of oculomotor nerve paralysis after endovascular therapy for posterior communicating aneurysm have been reported (19, 20). Iida et al. (10) reported a case of a patient with HFS caused by the intravascular treatment of the saccular aneurysm of the vertebral artery. The authors believed that the relief of HFS symptoms was due to the relief of aneurysm pulsation rather than the relief of compression. In this case, the direct electrophysiological monitoring of the aneurysm revealed that LSR disappeared immediately after aneurysm embolization, and the patient's spasm of the right hemifacial muscle was immediately relieved after surgery. The aneurysm volume did not decrease significantly after aneurysm embolization, but we discovered that LSR disappeared immediately after endovascular therapy, so we believe that aneurysm pulsation is important in HFS. However, when monitoring MVD, it has been observed that LSR disappears in some patients after opening the dura mater, releasing cerebrospinal fluid, and pulling the cerebellum. Thirumala et al. (3) hypothesized that the surgical procedure preceding the separation of the responsible vessels could alter the local anatomical relationship between the responsible vessels and the facial nerve, acting as a temporary decompression. As a result, whether there will be slight morphological changes after aneurysm endovascular treatment, resulting in changes in the occupying effect, needs to be confirmed further. Liu et al. (21) proposed that only a “just right” compression can generate and trigger the symptom. “Just right compression” means an appropriate compression with a certain specific frequency, amplitude, and angle. In fact, when PAO is conducted in this case, changes in some of the parameters of the compression, such as angiodynamics, will cause symptoms to halt. Simultaneously, the abnormal impulse generation would cease.

## 4. Limitation

The main limitation of this study is the lack of enough cases. We cannot provide controlled clinical research to demonstrate the availability and efficacy of our strategy. Following a direct procedure, such as MVD, HFS typically disappears right away. However, it takes time for HFS to recover after endovascular therapy (10). Although the symptoms completely disappeared after a month, a more-than-6-Month follow-up is needed. If the spasm recurs, open surgery such as a bypass or MVD could be an excellent choice.

## 5. Conclusion

HFS caused by a fusiform aneurysm of the vertebral artery is extremely rare. In this case, endovascular treatment can not only prevent aneurysm rupture but also successfully relieve HFS. Intraoperative monitoring of LSR can effectively predict postoperative HFS efficacy. The reason may be the disappearance of aneurysm wall pulsation after endovascular therapy, but the specific reason needs to be further verified. Endovascular therapy can be a safe and effective way to relieve HFS caused by compression of vertebral aneurysms.

## Data availability statement

The original contributions presented in the study are included in the article/Supplementary material, further inquiries can be directed to the corresponding author.

## Ethics statement

Written informed consent was obtained from the individual(s) for the publication of any potentially identifiable images or data included in this article.

## Author contributions

PH put forward research ideas and took the responsibility of communicating with the patient's family and obtaining the authorization of this study. HJ was responsible for drafting articles and revising the article. ZL was responsible for literature searches and final proofreading. All authors contributed to the article and approved the submitted version.

## Supplementary material

Supplementary Video 1 Patient had involuntary left facial spasm before Interventional operation.

Supplementary Video 2 On the first day after Interventional operation, the patient's left facial spasm were significantly relieved.

Supplementary Video 3 The patient's symptoms did not completely disappear.

Supplementary Video 4 The patient's symptoms completely resolved after 1 month.
